# The importance of physical education and the intention to be physically active as predictors of subjective well-being in secondary school adolescents

**DOI:** 10.3389/fspor.2026.1748124

**Published:** 2026-04-10

**Authors:** Marco Batista, Jorge Rojo-Ramos, Antonio Castillo-Paredes, Carmen Galán-Arroyo

**Affiliations:** 1 Polytechnic University of Castelo Branco, Castelo Branco, Portugal; 2 SPRINT—IPCB, Sport Physical Activity and Health Research & Innovation Center, Castelo Branco, Portugal; 3 Promoting a Healthy Society Research Group (PHeSO), Faculty of Sport Sciences, University of Extremadura, Cáceres, Spain; 4 Grupo AFySE, Investigación en Actividad Física y Salud Escolar, Escuela de Pedagogía en Educación Física, Facultad de Educación, Universidad de Las Américas, Santiago, Chile

**Keywords:** adolescence, behavioral intention, physical activity, physical education, subjective well-being

## Abstract

**Introduction:**

In recent years, concerns about adolescent mental health and declining physical activity levels have increased across European educational systems, including Portugal. Understanding how Physical Education (PE) and students' intention to remain physically active contribute to subjective well-being is therefore essential for guiding educational practices and health promotion strategies. Within this context, the present study aimed to analyze these relationships in a group of secondary school students in Portugal, with a view to proposing evidence-based recommendations. This study aimed to analyze the role of the importance attributed to Physical Education (PE) and the intention to be physically active as significant statistical predictors of subjective well-being (SWB) in Portuguese secondary school adolescents.

**Methods:**

The sample consisted of 560 students, aged between 16 and 19 years, from different regions of Portugal. Validated scales were used to measure the variables of interest: Importance of PE, Intention to Be Active, Positive and Negative Affect, and Life Satisfaction. The data were analyzed using descriptive statistics, Spearman correlations, Kruskal-Wallis and Mann-Whitney tests, linear regressions, and simple mediation models (Hayes' model 4).

**Results:**

The results indicated high levels of physical activity intention and life satisfaction, low levels of negative affect, and strong internal consistency in the scales used. The importance attributed to PE and the intention to be active were significantly associated with positive affect and life satisfaction, with intention partially mediating the relationship between the importance of PE and positive affect, and fully mediating the relationship with negative affect. No significant mediation was found for life satisfaction. Additionally, significant differences were observed across school years, highlighting the 11th year as a stage of greater emotional vulnerability.x

**Discussion:**

The findings suggest the need to reinforce the value of PE in the curriculum and to promote educational strategies that encourage regular physical activity as a way to enhance SWB in the school context.

## 1 Introduction

Subjective well-being (SWB) among adolescents has increasingly emerged as a key pillar for positive youth development, reflecting how young people evaluate their lives in terms of overall life satisfaction, and the presence of positive and negative affect (1). The school setting-particularly Physical Education (PE)-is a privileged domain for promoting both mental and physical health in adolescents (2), especially when combined with behavioral intention to lead a physically active life.

Subjective well-being is a multidimensional construct that includes both cognitive (life satisfaction) and emotional (positive and negative affect) evaluations of one's life (3). Among adolescents, SWB is associated with self-esteem, social support, perceived competence, and engagement in meaningful activities such as sports and physical activity (4).

Recent research has increasingly emphasized the role of school-based physical activity and Physical Education in promoting psychological well-being and mental health among adolescents. Evidence indicates that regular engagement in physical activity during adolescence is associated with improved emotional regulation, higher life satisfaction, and lower levels of anxiety and depressive symptoms (5, 6). Beyond these general associations, several studies have highlighted the importance of motivational and psychosocial mechanisms linking participation in physical activity with emotional well-being within educational settings (7, 8). More recent research continues to reinforce this relationship, indicating that structured physical exercise programs implemented in school contexts can significantly enhance psychological well-being and reduce stress and anxiety among adolescents (9), while broader epidemiological evidence also shows that higher levels of physical activity are associated with improved emotional regulation, reduced risk of depression and anxiety, and greater life satisfaction in youth populations (10–12). Regular physical activity-particularly in school contexts-can serve as a buffer against psychosocial risk factors such as stress, anxiety, and bullying (13). Taken together, these findings reinforce the relevance of examining how students' perceptions of Physical Education and their behavioral intentions toward physical activity relate to indicators of subjective well-being.

Physical Education has a unique potential to promote students' holistic well-being. Beyond developing motor skills and healthy lifestyles, PE fosters values such as cooperation, resilience, and self-esteem (14). The pedagogical context of PE allows students to experience motor success, personal control, and positive interpersonal relationships-all of which are strongly linked to SWB.

According to Galán-Arroyo, Polo-Campos et al. (15), perceived motor self-efficacy tends to decline across educational stages, particularly during upper secondary school, which may compromise engagement in physical activity and overall well-being. It is therefore essential to implement pedagogical approaches that promote success experiences and positive feedback, enhancing students' sense of competence and autonomy.

The intention to maintain an active lifestyle is a strong predictor of future physical activity behaviors, according to the Theory of Planned Behavior (16). This intention is influenced by behavioral beliefs, subjective norms, and perceived control, and is shaped by the school context and experiences within PE.

Studies show that adolescents with stronger intentions to be physically active report higher levels of psychological well-being, self-esteem, and emotional regulation (6). This relationship is especially significant during transitional educational phases such as upper secondary school, where academic and social demands increase.

Perceived motor competence not only influences adherence to physical activity, but also impacts physical self-concept and emotional well-being (17). Galán-Arroyo, Mayordomo-Pinilla et al. (18) found that self-perceived physical fitness moderates the relationship between motor self-efficacy and physical self-concept, promoting a more positive body image.

Additionally, Galán-Arroyo, Herreruela-Jara et al. (19) observed that motor self-efficacy can mitigate the negative effects of a high BMI on self-concept and well-being, highlighting the importance of individuals' subjective perception of their competencies.

The link between physical activity and social well-being has also been demonstrated in bullying research. Galán-Arroyo, Flores-Ferro et al. (13) showed that students with higher motor self-efficacy and engagement in PE reported less involvement in school bullying situations. Rojo-Ramos et al. (20) reinforce this by showing that motor self-efficacy is negatively associated with cyberbullying behaviors, acting as a protective mechanism. Thus, PE and the intention to be physically active contribute to safer and healthier school environments, promoting collective well-being.

In the Portuguese upper secondary education context, increasing concerns have emerged regarding adolescent sedentary behavior, declining levels of physical activity, and growing mental health complaints among young people. Although Physical Education (PE) is widely recognized as a relevant school subject for promoting active lifestyles and psychosocial well-being, empirical evidence examining how students' perceptions of the importance of PE and their intention to remain physically active relate to subjective well-being outcomes remains limited, particularly within the Portuguese educational system. Addressing this gap may provide relevant insights for educational policies and pedagogical practices aimed at strengthening the role of PE in promoting adolescent well-being.

Based on the literature reviewed, the present study aimed to:

1. Analyze the relationship between the perceived importance of Physical Education and subjective well-being among secondary school students;
2. Examine whether the intention to be physically active predicts subjective well-being indicators (positive affect, negative affect, and life satisfaction);
3. Explore whether the intention to be physically active mediates the relationship between the perceived importance of Physical Education and subjective well-being outcomes.

## 2 Material and methods

The study conducted is a cross-sectional correlational analysis aimed at assessing the state of a population at a specific point in time through the use of questionnaires and direct measurements applied to each observational unit (21). In terms of direct intervention related to the study topic, it is classified as an observational study of a descriptive nature. This is due to the absence of manipulation of independent variables (21), with the variables being presented as they occur naturally, without interference from the researchers.

### 2.1 Participants

The sampling method used in this study was purposive (22), as the aim was to target a specific stratum of the student population—students from the 10th to the 12th grade in Portuguese public secondary education, located in different regions of mainland Portugal and the islands. The participating schools included both urban and semi-urban educational settings, reflecting a diverse educational context within the Portuguese public school system.

The sample consisted of 560 students aged between 16 and 19 years (M = 17.06, σ  = 0.86), of whom 304 students (54.3%) identified as male and 256 students (45.7%) as female. All participants were enrolled in public secondary schools in Portugal, distributed as follows: 192 students (34.3%) in the 10th grade, 144 students (25.7%) in the 11th grade, and 224 students (40%) in the 12th grade.

Each student attended two weekly Physical Education sessions—one lasting 90 min and the other 60 min—totaling 150 min of PE per week. In addition to these PE classes, 48.6% of the students reported engaging in 150 to 180 min of physical activity during the week.

### 2.2 Instruments

The Validated Portuguese versions of the following construct variables were used for data collection:

Perceived Importance of Physical Education: The Physical Education Importance Scale (23) was used, consisting of three items (e.g., “I consider it important to have Physical Education classes”), translated into Portuguese. Responses were given on a 4-point Likert scale, ranging from 1 (“strongly disagree”) to 4 (“strongly agree”).

Intention to Be Physically Active: The Portuguese validated version of the *Intention to be Physically Active Scale* by Jiménez et al. (24) was used. The questionnaire comprises three items assessing a single factor—intention to be physically active (e.g., “I am interested in developing my physical fitness”).

Affect: To assess affect, the Positive and Negative Affect Schedule (25) was used. The scale consists of 20 items, with 10 adjectives assessing positive affect (e.g., interested, excited, strong, alert, enthusiastic) and the remaining 10 assessing negative affect (e.g., distressed, worried, guilty). Each item is rated on a 5-point Likert scale ranging from 1 (“very slightly or not at all”) to 5 (“extremely”).

Life Satisfaction: Life satisfaction was measured using the Satisfaction With Life Scale (SWLS) (26), which includes five items (e.g., “In most ways, my life is close to my ideal”). Responses are provided on a 7-point Likert scale ranging from 1 (“strongly disagree”) to 7 (“strongly agree”).

### 2.3 Procedures

The study was approved by the Ethics Committee of the Polytechnic University of Castelo Branco (Portugal). Written informed consent was obtained from all participants, including parental consent for underage students, in accordance with the ethical standards of the Declaration of Helsinki.

All participants were treated in compliance with the ethical standards of the American Psychological Association regarding informed consent, confidentiality, and anonymity. Written informed consent was obtained from all participants, including parental consent in the case of underage students.

The administration of the questionnaire packet—which included the previously described scales along with sociodemographic data—was conducted in the presence of the principal investigator. A brief explanation of the study's objectives, structure, and instructions for completion was provided. The principal investigator remained available throughout the process to address any questions or issues that arose.

The questionnaires were administered in paper format, and the average completion time was approximately ten minutes.

### 2.4 Data analysis

Data analysis was conducted using the Statistical Package for the Social Sciences (SPSS 23.0). Normality was assessed using the Kolmogorov–Smirnov test with Lilliefors correction, and the results indicated deviations from normality in all variables.

Descriptive statistics were then calculated based on the mean and standard deviation, and reliability indicators were assessed using Cronbach's alpha (27).

For inferential analysis, Spearman's bivariate correlation was performed. To compare groups, the Kruskal–Wallis test was applied, followed by the Mann–Whitney *U*-test for pairwise comparisons, in order to identify potential differences between groups. The significance level was set at 0.05, corresponding to a confidence level of at least 95%.

Additionally, we conducted mediation model analyses as proposed by Hayes (28), using Model 4 with bootstrapping procedures based on 5,000 samples.

## 3 Results

Five key variables were analyzed in this study: Perceived Importance of Physical Education, Intention to Be Physically Active, Positive Affect, Negative Affect, and Life Satisfaction. Table 1 presents the mean values (M), standard deviations (σ), internal consistency (Cronbach's α), Kolmogorov–Smirnov normality test results (KS), and Spearman correlations between variables.

**Table 1:** Descriptive statistics, normality test, and correlation between study variables.

Variable	M ± σ	α	KS	2	3	4	5
1. Importance of Physical Education	3.36 ± 0.57	0.74	<.01	0.38**	0.44**	0.06	0.29**
2. Intention to Be Physically Active	4.45 ± 0.73	0.90	<.01	–	0.57**	0.24**	0.23**
3. Positive Affects	3.59 ± 0.84	0.90	<.01		–	0.44**	0.46**
4. Negative Affects	1.94 ± 1.06	0.95	<.01			–	0.04
5. Life Satisfaction	4.93 ± 1.24	0.89	<.01				–

M ± σ—Mean ± standard deviation; α—cronbach alfa; KS, kolmogorov smirnov.

* *p* < .05.

** *p* < .01.

Overall, the descriptive results reveal high levels of intention to engage in physical activity (M = 4.45; σ = 0.73) and life satisfaction (M = 4.93; σ = 1.24), along with low levels of negative affect (M = 1.94; σ = 1.06). Positive affect showed a moderately high mean (M = 3.59; σ = 0.84), while the perceived importance of Physical Education was reported at moderate-to-high levels (M = 3.36; σ = 0.57). Taken together, these results indicate a generally favorable profile of subjective well-being among participants.

Regarding the internal reliability of the scales, high levels of internal consistency were observed across all variables, with Cronbach's alpha coefficients ranging from 0.74 (Importance of Physical Education) to 0.95 (Negative Affect), demonstrating the strong psychometric properties of the instruments used. Additionally, the Kolmogorov–Smirnov test results indicated violations of normality in the data distributions (*p* < .01), justifying the use of non-parametric analyses.

In terms of correlations between variables, a statistically significant correlation was observed between the importance attributed to Physical Education and the intention to be physically active (*r* = 0.38; *p* < .01), as well as with positive affect (*r* = 0.44; *p* < .01) and life satisfaction (*r* = 0.29; *p* < .01), though these relationships were of moderate to weak magnitude. The intention to be physically active showed moderate correlations with positive affect (*r* = 0.57; *p* < .01), and weaker correlations with negative affect (*r* = 0.24; *p* < .01) and life satisfaction (*r* = 0.23; *p* < .01).

In turn, positive affect was significantly correlated with life satisfaction (*r* = 0.46; *p* < .01) and negative affect (*r* = 0.44; *p* < .01), generally highlighting its important role in perceived well-being.

Interestingly, negative affect did not show a significant correlation with life satisfaction (*r* = 0.04; *p* > .05), which warrants critical reflection. This finding may suggest a partial dissociation between emotional distress and overall life evaluation, or it could reflect response bias or high intraindividual variability. This result highlights the need for a more detailed analysis of the differential role of positive and negative affect in the perception of well-being, possibly through mediation or moderation models.

Overall, the findings reveal consistent associations between affective-motivational variables and well-being indicators, reinforcing the importance of promoting positive attitudes toward Physical Education and physical activity as pathways to emotional balance and overall life satisfaction.

In Table 2 analysis using Kruskal–Wallis test revealed statistically significant differences between the different school years (10th, 11th, and 12th grades) regarding all variables analyzed: Importance of Physical Education (IPE), Intention to Be Physically Active (IPA), Positive Affect (PA), Negative Affect (NA), and Life Satisfaction (LS) (*p* < .01 for all variables). The Mann–Whitney test was then applied to determine between which groups these differences existed.

There was a progressive and statistically significant decline in the perceived importance of Physical Education as students progressed through secondary school:

Students in 10th grade attributed significantly more importance to Physical Education than those in 11th and 12th grades (*p* < .01). This pattern suggests a possible progressive devaluation of the subject as schooling progresses, a hypothesis that should be explored in future qualitative or longitudinal studies.

The results for intention to be physically active showed relatively high values across all grades, with statistically significant differences between groups.

Although intention remained high overall, 11th-grade students revealed significantly lower intentions than those in 10th and 12th grades. This downward and subsequent upward slope may reflect contextual variables associated with the academic workload of 11th grade, often considered the most demanding of high school.

10th-grade students showed significantly higher levels of positive affect compared to their peers in subsequent grades (*p* < .01). This could indicate a decrease in emotional well-being throughout school, possibly associated with increased academic pressure and anxiety related to career decisions. Regarding negative affect, 10th-grade students reported the highest levels (M = 2.15 ± 1.22), followed by 11th-grade students (M = 1.97 ± 0.99) and 12th-grade students (M = 1.75 ± 0.91). These differences were statistically significant between 10th-grade and 12th-grade students. Interestingly, this contradicts the trend observed for positive affect, suggesting a possible emotional duality in 10th-grade students, who appear to experience greater intensity of both positive and negative emotions simultaneously.

The variable “satisfaction with life” presents overall high values, with no substantial variations between the groups:

The differences were statistically significant between 10th-grade and 11th-grade students, with life satisfaction being slightly higher at the extremes (10th-grade and 12th-grade) compared to 11th-grade students. This data may reinforce the interpretation that the 11th year constitutes a particularly challenging and critical moment in the school career.

The results of the linear regression analyses shown in Table 3 reveal that both the importance attributed to Physical Education and the intention to be physically active are statistically significant predictors of several dimensions of participants' subjective well-being, namely positive affect, negative affect, and life satisfaction.

**Table 2:** One-way analysis of dependent variables as a function of school grade students.

Variable M ± σ	IPE**	IPA**	PA**	NA**	LS**
10th grade (a)	3.63 ± 0.35^b^^,^^c^	4.50 ± 0.75^b^^,^^c^	4.09 ± 0.64^b^^,^^c^	2.15 ± 1.22^c^	5.03 ± 1.34^b^^,^^c^
11th grade (b)	3.35 ± 0.54^a^^,^^c^	4.28 ± 0.80^a^	3.27 ± 0.71^a^^,^^c^	1.97 ± 0.99	4.79 ± 1.10^a^^,^^c^
12th grade (c)	3.14 ± 0.64^a^^,^^b^	4.51 ± 0.64^a^	3.38 ± 0.88^a^^,^^b^	1.75 ± 0.91^a^	4.95 ± 1.24^a^^,^^b^

M ± σ -Mean ± Standard deviation; IPE, importance of physical education; IPA, intention to be physically active.

PA, positive affects; NA, negative affects; LS, life satisfaction; Kruskal–Wallis.

^a^10° Grade, ^b^11° grade, ^c^12° grade

* *p* < .05.

** *p* < .01.

**Table 3:** Linear regression of the variables: importance of physical education and intention to be physically active as predictors of positive affect, negative affect, and life satisfaction.

Predictor Variable	Beta	*t*	*p*	*R* ^2^
Positive affects				
Importance of Physical Education	0.46	12.37	<0.001**	0.21
Intention to Be Physically Active	0.54	15.18	<0.001**	0.29
Negative affects				
Importance of Physical Education	0.12	2.90	0.004**	0.01
Intention to Be Physically Active	0.26	6.33	<0.001**	0.07
Life satisfaction				
Importance of Physical Education	0.25	5.99	<0.001**	0.06
Intention to Be Physically Active	0.13	3.20	0.001**	0.02

* *p* < .05.

** *p* < .01.

The importance of Physical Education proved to be a positive and statistical significant predictor of positive affect (*β* = 0.46, *t* = 12.37, *p* < 0.001), explaining 21% of the variance (*R*^2^ = 0.21). The intention to be physically active revealed an even more pronounced effect (*β* = 0.54, *t* = 15.18, *p* < 0.001), explaining 29% of the variance in positive affect. These results indicate that young people who value Physical Education more and demonstrate greater intention to remain physically active tend to experience higher levels of positive emotions. In the case of negative affects, the importance of Physical Education showed a modest but significant positive relationship (*β* = 0.12, *t* = 2.90, *p* = 0.004), with a very small *R*^2^ value (0.01), suggesting a limited explanatory contribution.

The intention to be physically active, although also positive (*β* = 0.26, *t* = 6.33, *p* < 0.001), showed a slightly more expressive effect, explaining 7% of the variance. Although both predictors were statistically significant, the direction of the association is somewhat counterintuitive, suggesting that higher levels of intention and appreciation for PE may also coexist with higher (albeit residual) levels of negative affects.

This apparent contradiction warrants further exploration and may suggest the existence of moderating variables (e.g., academic stress, pressure for physical or physical performance) that attenuate or reverse the expected positive impact. Regarding life satisfaction, the importance attributed to Physical Education appears again as a positive and significant statistical predictor (*β* = 0.25, *t* = 5.99, *p* < 0.001), with an explanatory contribution of 6% (*R*^2^ = 0.06). The intention to be physically active also positively predicts this variable (*β* = 0.13, *t* = 3.20, *p* = 0.001), although with less explanatory strength (*R*^2^ = 0.02). These findings suggest that, although both variables are positively associated with the perception of life satisfaction, the appreciation of the Physical Education discipline seems to have greater weight in predicting this dimension.

**Figure 1:**
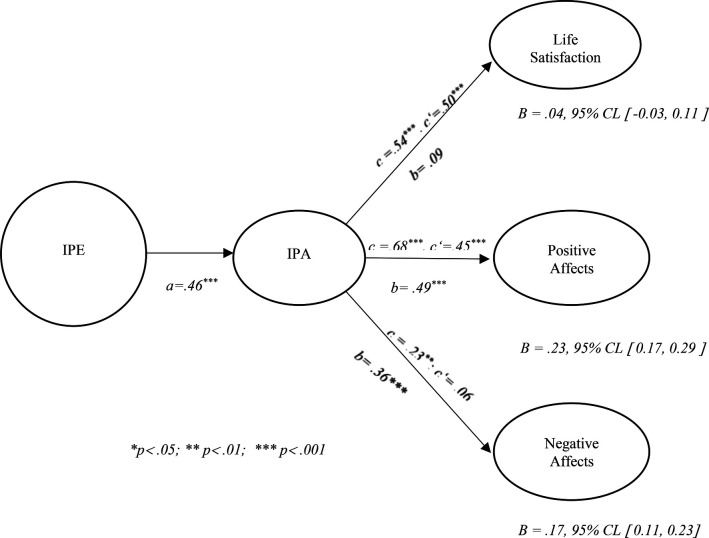
Mediation models between the importance of physical education (X) and subjective well-being variables (Y) mediated by the intention to be physically active (M).

Figure 1 shows the simple mediation models, where three simple mediation analyses were conducted based on PROCESS Model 4 (28). Importance of Physical Education (IPE) was considered the statistical predictor variable (X), Intention to Be Physically Active (IPA) was the mediator variable (M), and the subjective well-being outcomes were: Life Satisfaction (LS), Positive Affect (PA), and Negative Affect (NA) (Y).

1. Mediation between IPE and Life Satisfaction (LS)

The total effect of IPE on LS was significant, c = 0.54, *p* < .001. However, the direct effect after the introduction of the IPA mediator became non-significant (c' = 0.50, *p* > .05), with an indirect path (a × b) of B = 0.04, 95% CI [−0.03, 0.11]. The indirect effect was not statistically significant, as the confidence interval included zero, indicating no evidence of a statistically significant mediation effect.

2. Mediation between IPE and Positive Affect (PA)

The total effect was significant (c = 0.68, *p* < .001) and significantly reduced after the inclusion of IPA as a mediator (c' = 0.45, *p* < .001), indicating partial mediation. The indirect effect, expressed by B = 0.23, 95% CI [0.17, 0.29], indicates a statistically significant mediation effect, with IPA partially mediating the relationship between IPE and positive affect. 3. Mediation between IPE and Negative Affect (NA).

The total effect of IPE on NA was significant and negative (c = −0.23, *p* < .01). After the introduction of IPA, the direct effect became non-significant (c' = −0.06, *p* > .05), suggesting full mediation. The indirect effect was B = 0.17, 95% CI [0.11, 0.23], indicating a statistically significant mediation effect.

Summing up the results of the mediation models, they show that Intention to Be Physically Active significantly mediates the relationship between the Importance attributed to Physical Education and positive and negative affects, with partial mediation in the former case and full mediation in the latter. However, this mediation was not significantly verified in life satisfaction, indicating that this outcome may depend on factors other than physical activity intention.

## 4 Discussion

The results of this study generally indicate a favorable profile of subjective well-being among participants. There were high levels of intention to be physically active and life satisfaction, accompanied by low levels of negative affect and moderately high levels of positive affect. These findings corroborate previous research that associates active lifestyles with better emotional and mental health indicators in adolescents (5).

From a psychometric perspective, all scales used demonstrated adequate levels of internal consistency, with Cronbach's *α* coefficients between 0.74 and 0.95, in line with the theoretical indicators proposed by Nunnally (27).

Correlations between variables revealed significant associations between the importance attributed to Physical Education, intention to be physically active, and indicators of subjective well-being. In particular, physical activity intention showed moderate correlations with positive affect and weaker correlations with life satisfaction and negative affect. These data point to the importance of motivational engagement in young people's emotional experience.

Interestingly, there was no significant correlation between negative affect and life satisfaction, suggesting that these dimensions may function relatively independently. This dissociation is in line with two-dimensional models of well-being (1), which posit the existence of distinct mechanisms underlying positive and negative experiences.

Group comparisons using the Kruskal–Wallis test revealed significant differences between grade levels in all variables studied. Tenth-grade students attributed greater importance to PE and reported higher levels of positive affect compared to their 11th- and 12th-grade counterparts. This trend may reflect a progressive decline in the value of PE throughout secondary school, possibly associated with increased academic pressure and the centrality of nationally-examined subjects (29). The 11th grade stood out as a particularly critical period, with lower scores for intention to exercise, positive affect, and life satisfaction. This pattern can be interpreted in light of the increased academic demands of this school year, often referred to as the most demanding of high school.

Regarding negative affect, there was a pattern opposite to that of positive affect: 10th grade students presented the highest scores, followed by 11th and 12th graders. This coexistence of positive and negative emotions in the same age group may indicate an ambivalent affective response to the school context, reflecting both enthusiasm and stress.

Given the cross-sectional design of this study, the observed predictive relationships should be interpreted with caution, as causal inferences cannot be established. Regression analyses indicated that both the importance attributed to Physical Education and the intention to be physically active were statistically associated with positive affect, negative affect, and life satisfaction. These relationships should be interpreted as statistical associations rather than causal effects. Physical activity intention revealed greater explanatory power for positive affect (*R*^2^ = 0.29) and negative affect (*R*^2^ = 0.07), while the importance of physical activity proved more relevant in predicting life satisfaction (*R*^2^ = 0.06).

Although several associations reached statistical significance, the proportion of explained variance observed in some models was relatively modest. For instance, the importance attributed to Physical Education accounted for only a small proportion of the variance in negative affect, suggesting that this relationship should be interpreted cautiously. Subjective well-being in adolescence is widely recognized as a multifactorial construct influenced by a broad constellation of contextual, social, and psychological variables, including family environment, peer relationships, academic experiences, and perceived competence (1, 4). From this perspective, the results should not be interpreted as indicating that Physical Education alone plays a determinant role in adolescents' well-being, but rather that it represents one relevant component within a wider network of influences shaping young people's emotional experiences.

The positive relationship between physical activity intention and negative affect, although modest, appears counterintuitive and may be associated with contextual variables, such as stress associated with physical performance, aesthetic pressure, or social comparison. One possible explanation for this counterintuitive association may lie in the discrepancy between intention and actual behavior. Adolescents who report stronger intentions to be physically active may simultaneously experience frustration when academic demands or contextual constraints limit their opportunities to engage in physical activity. Additionally, performance-related pressure, body image concerns, and social comparison processes common during adolescence may contribute to higher emotional tension among students who are more involved in physical activity contexts. These results suggest the need to integrate moderating variables in future studies, which may explain unexpected variations in the associations.

Mediation analyses performed with PROCESS model 4 (28) allowed us to further explore the mediating role of physical activity intention in the relationship between the importance attributed to physical activity and subjective well-being outcomes. The results show significant mediation:

- Positive affects: significant partial mediation, with a reduction in the direct effect after the introduction of the mediator (IPE → IPA → PA).
- Negative affects: significant total mediation, suggesting that the impact of IPE on negative affects occurs exclusively through intention to practice.
- Life satisfaction: no significant mediation was observed, indicating that factors other than physical activity intention may influence this dimension.

From a theoretical perspective, these findings highlight the role of behavioral intention as a psychological mechanism linking students' perceptions of Physical Education with their emotional experiences. However, the absence of mediation for life satisfaction suggests that broader life evaluations are likely shaped by a wider set of contextual influences beyond school-based physical activity. These data reinforce the importance of behavioral intention as a key variable in young people's emotional experience, but also suggest that global well-being (assessed by life satisfaction) depends on a broader and more complex network of factors (4, 30).

Any study is not without its limitations. The cross-sectional nature of the study prevented the establishment of causal relationships between the variables analyzed. Although statistically significant associations were identified, it is not possible to affirm that the perception of PE or the intention to be active directly causes changes in adolescents' subjective well-being.

Data were collected exclusively through self-report questionnaires, which may have introduced social desirability or subjective perception bias. Students may have tended to report behaviors or attitudes considered more acceptable or desirable.

The sample was selected for convenience and is restricted to a limited number of schools in Portugal. This aspect may limit the generalization of the results to other school contexts or regions of the country.

Relevant contextual variables such as family influence, socioeconomic environment, and the quality of Physical Education classes were not considered in this study and may directly or indirectly impact levels of Subjective Well-Being.

The results suggest several educational and research implications. First, it is essential to value Physical Education not only as a curricular subject, but also as a promoter of students' emotional and social well-being. Pedagogical strategies that foster intrinsic motivation, active participation, and perceived competence can enhance the positive effects identified.

The declining trend in the appreciation of Physical Education and positive affect requires curricular attention. It is suggested that pedagogical strategies that promote intrinsic motivation and continued engagement be strengthened, especially in the final years of high school.

The intention to engage in physical activity remains high, which may indicate a dissonance between attitude and perception of the subject, something that should be explored qualitatively or through cluster analysis.

The mixed emotional profile of 10th-grade students (high positive and negative affect) raises the hypothesis of greater emotional reactivity during this transition phase, which should be accompanied by psychological support interventions and the promotion of socio-emotional skills. The 11th grade appears to be a critical point in multiple variables (reduction in positive affect, lower life satisfaction, lower intention to be physically active) and should be considered a priority target for interventions that promote well-being and regular physical activity.

From a research perspective, it is recommended to include contextual variables (e.g., frequency of physical activity, motivation, social support) and adopt longitudinal models that allow for monitoring the evolution of variables over time. Qualitative studies can also provide a deeper understanding of students' perceptions and motivations regarding physical activity and the subject of Physical Education.

The findings of this study suggest that the perceived importance of Physical Education and the intention to be physically active are positively associated with indicators of subjective well-being among secondary school adolescents. However, these associations should be interpreted cautiously, as the explained variance in some models was modest and the cross-sectional design does not allow causal inferences. Rather than representing determining factors, these variables should be understood as contributing elements within the broader network of psychosocial influences shaping adolescent well-being.

There were also significant differences between school years, with 11th grade standing out as a period of greater emotional vulnerability, possibly associated with increased academic demands. These data highlight the importance of differentiated educational strategies and psychological support tailored to the specificities of each stage of the school trajectory.

Mediation analyses revealed that the intention to be physically active significantly mediates the relationship between the importance attributed to Physical Education and affect (positive and negative), but not life satisfaction. This finding highlights the need to integrate additional variables, particularly contextual and psychosocial variables, into explanatory models of young people's overall well-being.

As practical implications for physical education, from an applied perspective, the findings suggest that Physical Education teachers may play a central role in promoting students' psychological well-being by fostering positive attitudes toward physical activity and strengthening behavioral intention. Pedagogical strategies that emphasize autonomy-supportive teaching, inclusive participation, and mastery-oriented motivational climates may enhance students' engagement and emotional experiences in PE classes. Additionally, given the vulnerability identified among 11th-grade students, targeted interventions promoting active lifestyles and stress management could be integrated into Physical Education programs during this stage of schooling.

Future research should adopt longitudinal designs and incorporate additional contextual variables in order to better understand the complex mechanisms linking Physical Education, physical activity engagement, and adolescents' psychological well-being.

## Data Availability

The raw data supporting the conclusions of this article will be made available by the authors, without undue reservation.
